# Survival analysis in lung cancer patients with interstitial lung disease

**DOI:** 10.1371/journal.pone.0255375

**Published:** 2021-09-07

**Authors:** Hassan Alomaish, Yee Ung, Stella Wang, Pascal N. Tyrrell, Saly Abo Zahra, Anastasia Oikonomou

**Affiliations:** 1 Department of Medical Imaging, Sunnybrook Health Sciences Centre, University of Toronto, Toronto, Canada; 2 Department of Radiation Oncology, Sunnybrook Health Sciences Centre, University of Toronto, Toronto, Canada; 3 Department of Medical Imaging, University of Toronto, Toronto, Canada; 4 Department of Statistical Sciences, University of Toronto, Toronto, Canada; 5 Institute of Medical Sciences, University of Toronto, Toronto, Canada; Helmholtz-Zentrum Munich, GERMANY

## Abstract

**Objective:**

Lung cancer patients with interstitial lung disease (ILD) are prone for higher morbidity and mortality and their treatment is challenging. The purpose of this study is to investigate whether the survival of lung cancer patients is affected by the presence of ILD documented on CT.

**Materials and methods:**

146 patients with ILD at initial chest CT were retrospectively included in the study. 146 lung cancer controls without ILD were selected. Chest CTs were evaluated for the presence of pulmonary fibrosis which was classified in 4 categories. Presence and type of emphysema, extent of ILD and emphysema, location and histologic type of cancer, clinical staging and treatment were evaluated. Kaplan-Meier estimates and Cox regression models were used to assess survival probability and hazard of death of different groups. P value < 0.05 was considered significant.

**Results:**

5-year survival for the study group was 41% versus 48% for the control group (log-rank test p = 0.0092). No significant difference in survival rate was found between the four different categories of ILD (log-rank test, p = 0.195) and the different histologic types (log-rank test, p = 0.4005). A cox proportional hazard model was used including presence of ILD, clinical stage and age. The hazard of death among patients with ILD was 1.522 times that among patients without ILD (95%CI, p = 0.029).

**Conclusion:**

Patients with lung cancer and CT evidence of ILD have a significantly shorter survival compared to patients with lung cancer only. Documenting the type and grading the severity of ILD in lung cancer patients will significantly contribute to their challenging management.

## Introduction

Lung cancer is the second most common cancer in both men and women (not counting skin cancer). Lung cancer is the leading cause of death from cancer making up almost 25% of all cancer deaths. Mortality from lung cancer is high due to its frequent presentation at a late stage [1]. According to the 2020 statistics by the American Cancer Society, 228,820 new lung cancer cases will be diagnosed in 2020 and there will be 135,720 deaths due to lung cancer in USA. Each year more people die of lung cancer than of colon, breast and prostate cancers combined.

Patients with interstitial lung disease (ILD) are known to be at increased risk of developing lung cancer and more prone for higher morbidity and mortality [2–4]. The prevalence of lung cancer in patients with idiopathic pulmonary fibrosis (IPF) is estimated around 13.5% [5]. The treatment of lung cancer in these patients may be challenging, because intensive treatments, including surgery, radiation therapy, and chemotherapy, may induce iatrogenic acute exacerbation of the underlying pulmonary fibrosis or pneumonia that may result in significant complications and increased mortality.

Previous studies have reported lower survival rate in patients with lung cancer and IPF or combined pulmonary fibrosis and emphysema (CPFE), however fibrotic lung disease initially documented on chest CT may have different patterns from the typical UIP CT pattern [6, 7]. The coexistence of lung cancer and fibrotic lung disease may also impact survival of these patients and have dismal prognosis compared to patients with lung cancer only. According to our knowledge the survival outcomes of patients with lung cancer and fibrotic lung disease documented on chest CT according to the recent guidelines for the diagnosis of IPF have not been previously reported [8].

The purpose of this study is to investigate whether the survival of lung cancer patients is affected by the presence of concomitant ILD documented on chest CT. Secondary objectives of the study include the investigation of the prevalence and different CT patterns of ILD in patients with lung cancer as well as the histologic types and other characteristics of lung cancer in these patients.

## Materials and methods

This retrospective study was approved by the Research Ethics Board of Sunnybrook Health Sciences Centre (REB 357–2018). The Research Ethics Board waived the consent form.

### Patients

All patients with lung cancer diagnosed during the period between January 2010 and December 2016 at our institution were retrospectively collected from the registry.

Patients who were diagnosed with any other malignancy additional to lung cancer were excluded except for patients with cervical carcinoma in situ and non-melanoma skin cancer.

4670 lung cancer patients with available chest CT at initial staging were retrospectively identified. Patients with sarcoidosis, known history of pneumoconiosis or radiation therapy affecting the lungs and medical treatment with agents known to cause pulmonary fibrosis were excluded. 150 patients were identified as having signs of ILD on the baseline chest CT. Four out of the 150 patients with lung cancer and fibrosis were excluded as they could not be matched with patients in the control group (1 patient with stage IIB spindle cell carcinoma, 2 patients with stage IIA small cell carcinoma and 1 patient with stage IIIB small cell carcinoma). Finally, 146 patients were included in this case control study.

### Evaluation of pulmonary fibrosis on chest CT

Chest CTs were not performed with a standard protocol as they were done in multiple different institutions, however all of them were performed with thin slices (1–3 mm). A high resolution algorithm was occasionally applied.

The chest CTs were evaluated in consensus by two cardiothoracic imaging fellows and a thoracic radiologist with 18 years of experience in thoracic imaging for the presence of ILD on chest CT at initial diagnosis.

**Fig 1:**
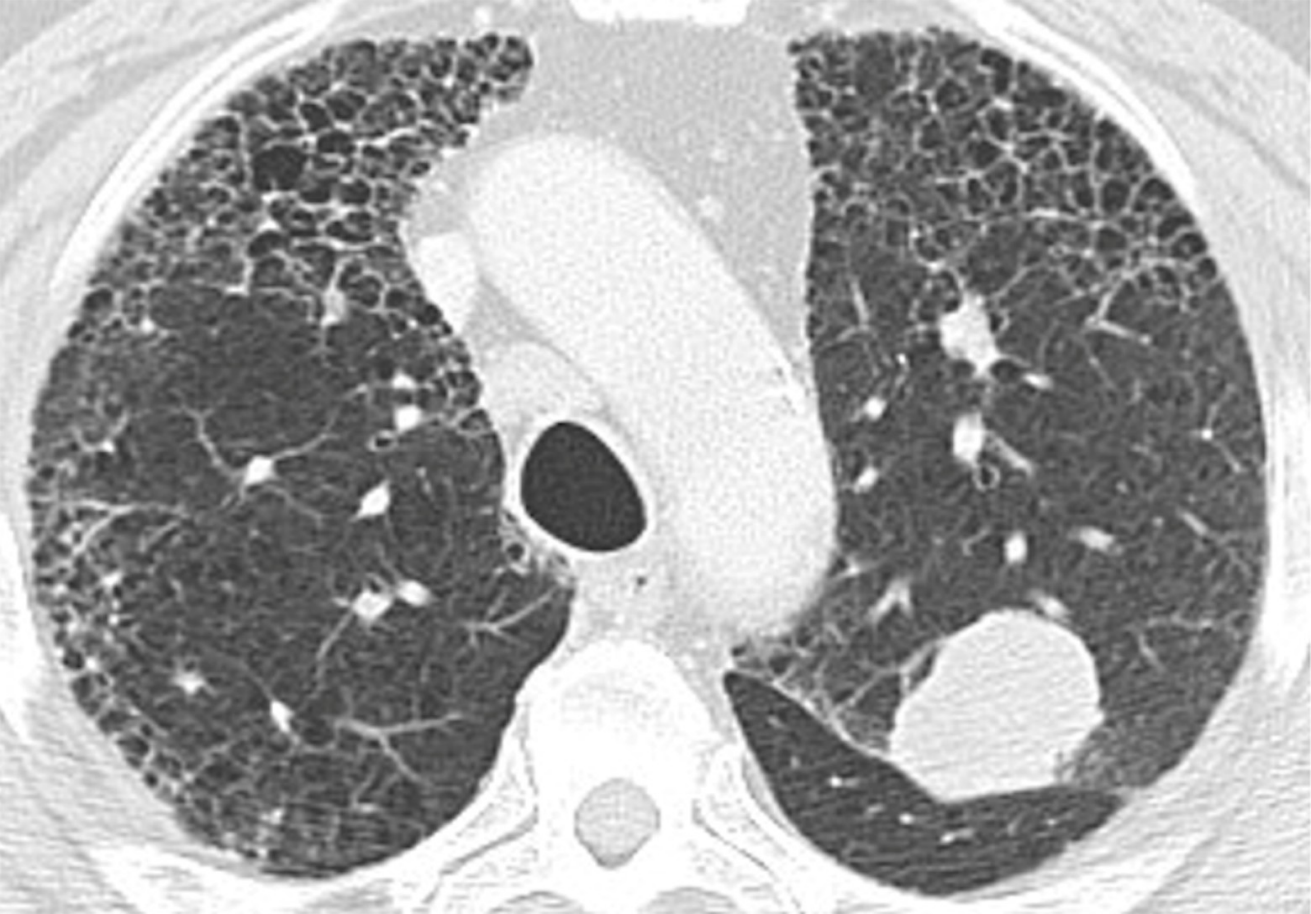
Squamous cell carcinoma and CT pattern typical for UIP. Axial CT image at the level of the upper lobes shows a left upper lobe squamous cell carcinoma and CT features of typical UIP.

**Fig 2:**
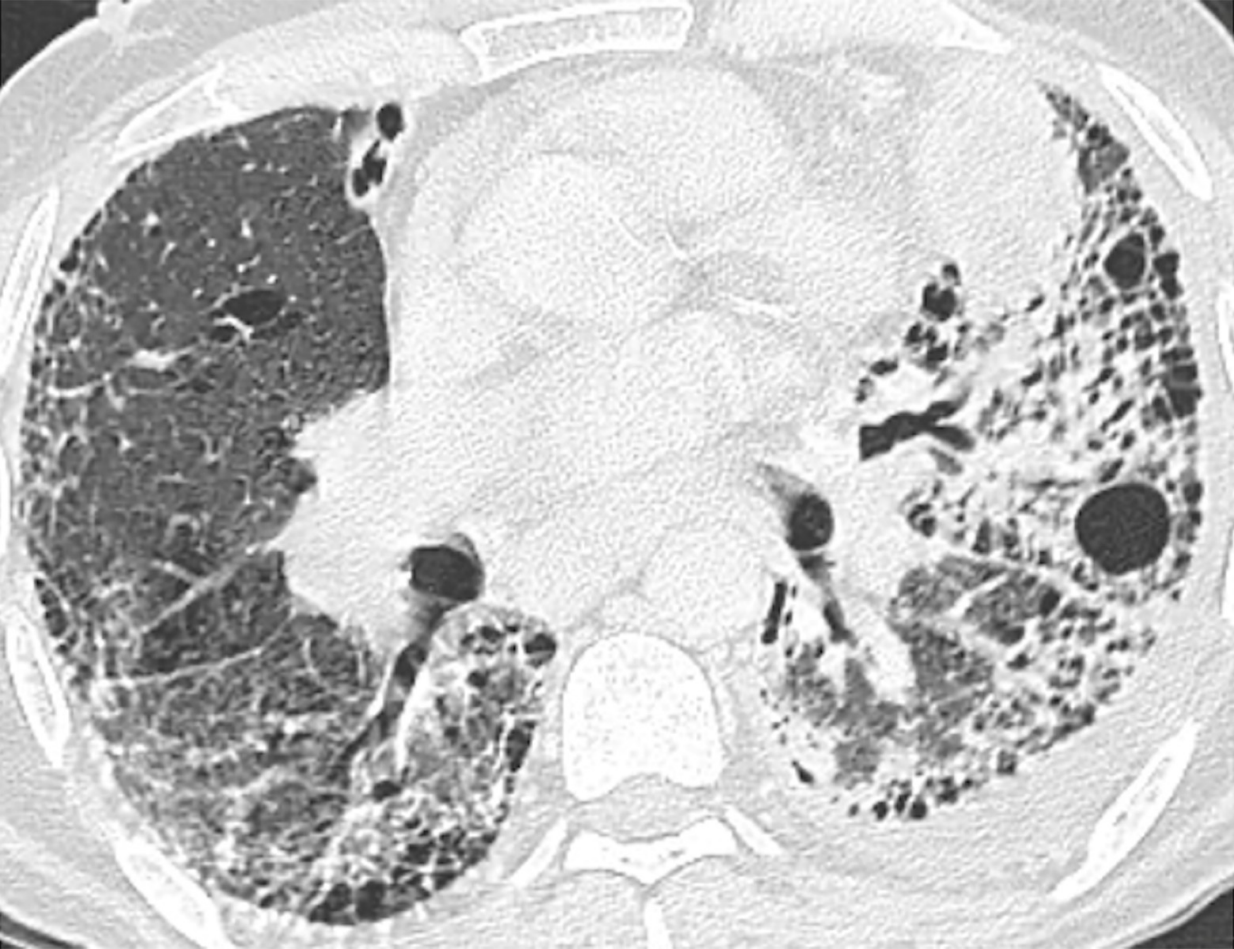
Lung cancer and CT pattern typical for UIP. Axial CT image at the level of the middle lobe and lingula demonstrates a lingular lung cancer (stage IV) and CT features consistent with typical UIP (traction bronchiectasis and subpleural honeycombing).

ILD was considered to be present if there was subpleural or peribronchovascular reticulation characterized by traction bronchiectasis/bronchiolectasis with or without surrounding ground glass opacities and with or without honeycombing [9]. The ILD was classified according to the following 4 different categories of CT pattern [8]: a) typical UIP, b) probable UIP, c) indeterminate for UIP and d) inconsistent with idiopathic pulmonary fibrosis (IPF) (Figs 1 and 2). To assess severity and degree of fibrosis, we considered both lungs as a whole and visually classified the extent of fibrosis into four different grades: a) mild <25%, b) moderate 25–49%, c) severe 50–74% and d) extensive >75%.

### Evaluation of emphysema on chest CT

**Fig 3:**
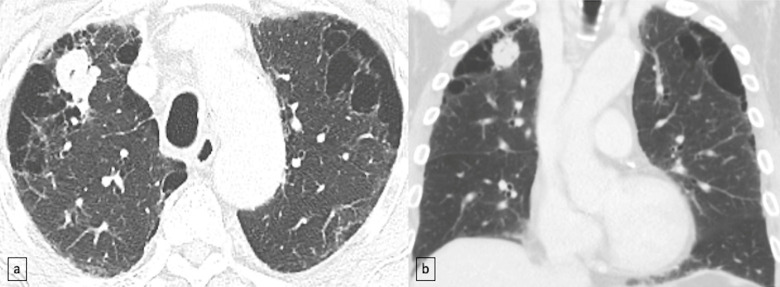
Squamous cell carcinoma and paraseptal emphysema. Axial (a) and coronal (b) CT images demonstrate right upper lobe squamous cell carcinoma and severe upper lobe paraseptal emphysema.

We classified the emphysema into the following 3 categories: paraseptal (PSE), centrilobular (CLE) and combined type (both paraseptal and centrilobular) (Fig 3). To grade the extent of emphysema, we used a visual modified grading system based on Fleischner classification resulting in the following 3 categories [10]: 1) Mild: trace CLE <0.5, mild CLE 0.5–5% and mild PSE <1cm, 2) Moderate: moderate CLE >5%, 3) Severe: confluent CLE, advanced destructive emphysema and substantial PSE >1cm).

### Location of lung cancer on CT

Location of the primary lung cancer was recorded according to the presence of the cancer in any of the 6 lung lobes (lingula was considered a separate lobe). The central or peripheral location of cancer was evaluated as follows: central location was considered if the cancer was seen in the inner 2/3 of lung parenchyma on axial plane and peripheral location was considered if the cancer was seen in the outer third of lung parenchyma on axial plane.

### Clinical data

Information regarding gender, age at diagnosis, date of diagnosis, smoking status, emphysema, fibrosis, cancer histology, clinical staging, presence and type of treatment and survival (recorded as time interval in months between the date of diagnosis and either date of death or last follow up of the patient), were also recorded from patients’ medical charts and institution’s registry. Patients were staged at initial diagnosis based on the 7^th^ edition of the TNM system, issued by IASLC (International Association for the Study of Lung Cancer) [11].

### Statistical analysis

Data were described as frequencies with percentage, medians with range, and means with standard deviation (SD) as appropriate. Randomization method for selecting a control group was applied using 3 variables (the patient’s gender, cancer histology and clinical staging). Individual case-control matching by gender, histologic cancer type and clinical staging was performed.

Median age was compared between groups by Mann- Whitney U test. The association between presence of ILD and categorical variables including gender, active smokers, ex-smokers, never smokers, presence of emphysema and death were measured by Chi-squared test. The association of clinical staging and type of histology was analyzed with the Fischer’s exact test.

The survival probability for the study and control groups during the study period was determined using Kaplan-Meier estimates. Survival time was calculated from the time of lung cancer diagnosis to the time of death or last follow-up. Survival curves between different groups were compared using the log-rank test.

Cox regression models were used to assess hazard of death of different groups and other risk factors. Differences in survival between the study and control groups were also assessed according to histologic type of cancer, clinical stage, presence and type of treatment, type and grade of fibrosis, type and grade of emphysema, gender and smoking status. P value less than 0.05 was considered significant. Statistical analyses were performed using SAS statistical software for windows 9.4 (SAS Institute Inc., Cary, NC USA).

## Results

### Patients’ characteristics

114 (78%) out of 292 patients included in the study were male (146 in the study and control group respectively). Median and average age at diagnosis of lung cancer in the study group was 76 years (range: 53–94) and in the control group was 73 and 71 years respectively (range: 36–92).

In the study group 50 patients were active smokers (34%), 70 patients were ex-smokers (48%), 10 patients had never smoked (7%) and 16 patients had unknown smoking status (11%).

In the control group 51 patients were smokers (35%), 54 patients had stopped smoking (37%), 12% of the patients had never smoked and 16% of the patients had unknown smoking status (Table 1).

**Table 1:** Patients’ characteristics.

Group Variables	Patients with Lung Cancer and ILD	Patients with Lung Cancer only	P-value
Median age (range)	76 (53–94)	73 (36–92)	0.0004
Gender–male, n (%)	114 (78)	114 (78)	1.0000
Active smokers, n (%)	50 (34)	51 (35)	1.0000
Ex-smokers, n (%)	70 (48)	54 (37)	0.0582
Never smokers, n (%)	10 (7)	17 (12)	0.1094
Emphysema, n (%)	117 (80)	103 (71)	0.0573
Death, n (%)	70 (48)	54 (37)	0.0582
5-year survival	41%	48%	0.0092
Treatment, n (%)			0.0006
• Radiation	74 (51)	88 (60)	
• Chemotherapy	9 (6)	3 (2)	
• Both	22 (15)	37 (25)	
• No treatment/unknown	41 (28)	18 (12)	

### Histology and clinical staging

Squamous cell carcinoma (SCC) was the most common histologic type of cancer seen in 67 patients (46%) followed by adenocarcinoma seen in 39 patients (27%) (Fig 1). SCC was the most common type of lung cancer in patients with CT pattern “typical for UIP”, “probable UIP” and “indeterminate for UIP”. However, in patients with CT pattern “inconsistent with IPF” the most common histologic type was adenocarcinoma in 9 cases (50%) followed by SCC in 6 cases (33%). There were 14 cases of small cell carcinoma (10%), 11 cases of carcinoma-not otherwise specified (7%), 8 cases of non-small cell carcinoma (5%) and 4 cases of large cell carcinoma (3%). A single case of sarcoma (1%) and 2 cases of carcinoid tumor (1%) were also included in the study.

The vast majority of patients were at advanced clinical staging (stage IIIA and IV) at the time of diagnosis (Table 2).

**Table 2:** Number of patients per histologic type of cancer and clinical staging.

Staging Cancer type	IA	IB	IIA	IIB	IIIA	IIIB	IV	p‐value
Squamous	8	8	1	5	18	6	21	<0.0001
Adenocarcinoma	4	4	0	2	10	3	16	
Small cell carcinoma	0	0	0	0	3	1	10	
Large cell carcinoma	0	0	0	1	0	1	2	
NSCLC	1	0	0	1	3	0	3	
NOS	5	2	0	0	0	0	4	
Sarcoma	0	0	0	0	0	1	0	
Carcinoid	0	0	0	0	0	0	2	

### Classification and extent of ILD and emphysema

According to the classification of ILD on chest CT, 51 patients had typical for UIP CT pattern (35%), 44 patients had probable UIP CT pattern (30%), 33 patients had indeterminate for UIP CT pattern (23%) and 18 patients had a CT pattern inconsistent with IPF diagnosis (12%).

According to the grading system for the extent of ILD on chest CT, 90 patients were found to have mild fibrotic changes (61.6%), 47 patients had moderate (32.2%) and 9 patients had severe fibrotic changes (6.2%) (Table 3).

**Table 3:** Classification and extent of ILD and extent of emphysema on CT in the study group.

	Patients, n (%)
Typical for UIP	51/146 (35)
Probable for UIP	44/146 (30)
Indeterminate for UIP	33/146 (23)
Not consistent with UIP	18/146 (12)
ILD—Grade I (mild)	90/146 (62)
ILD—Grade II (moderate)	47/146 (32)
ILD—Grade III (severe)	9/146 (6)
Emphysema–mild	45/117 (39)
Emphysema—moderate	27/117 (23)
Emphysema–severe	45/117 (39)

117 patients of the study group had emphysema on CT (80%) compared to 104 of the control group (71%). In the study group 45 patients had mild and severe emphysema accordingly (39%) and 27 had moderate emphysema (23%) (Table 3).

### Location of lung cancer on CT

Lung cancer was more commonly found in peripheral location involving the areas of fibrosis: in 32 cases in the “typical for UIP” group (62%), in 29 cases in the “probable UIP” group (67%), in 23 cases in the “indeterminate UIP” group (68%) and in 10 cases in the “inconsistent with IPF” group (59%).

The lung cancers were more commonly located in the right lower lobe in both “UIP” (21%) and “indeterminate UIP” groups (26%) and in the right upper lobe in “probable UIP” group 1 (24.5%) followed by RLL (22.5%). However, in patients with “inconsistent with IPF” CT pattern the most common location was the right upper lobe (41%) followed by left upper lobe (35%).

### Survival analysis

**Fig 4:**
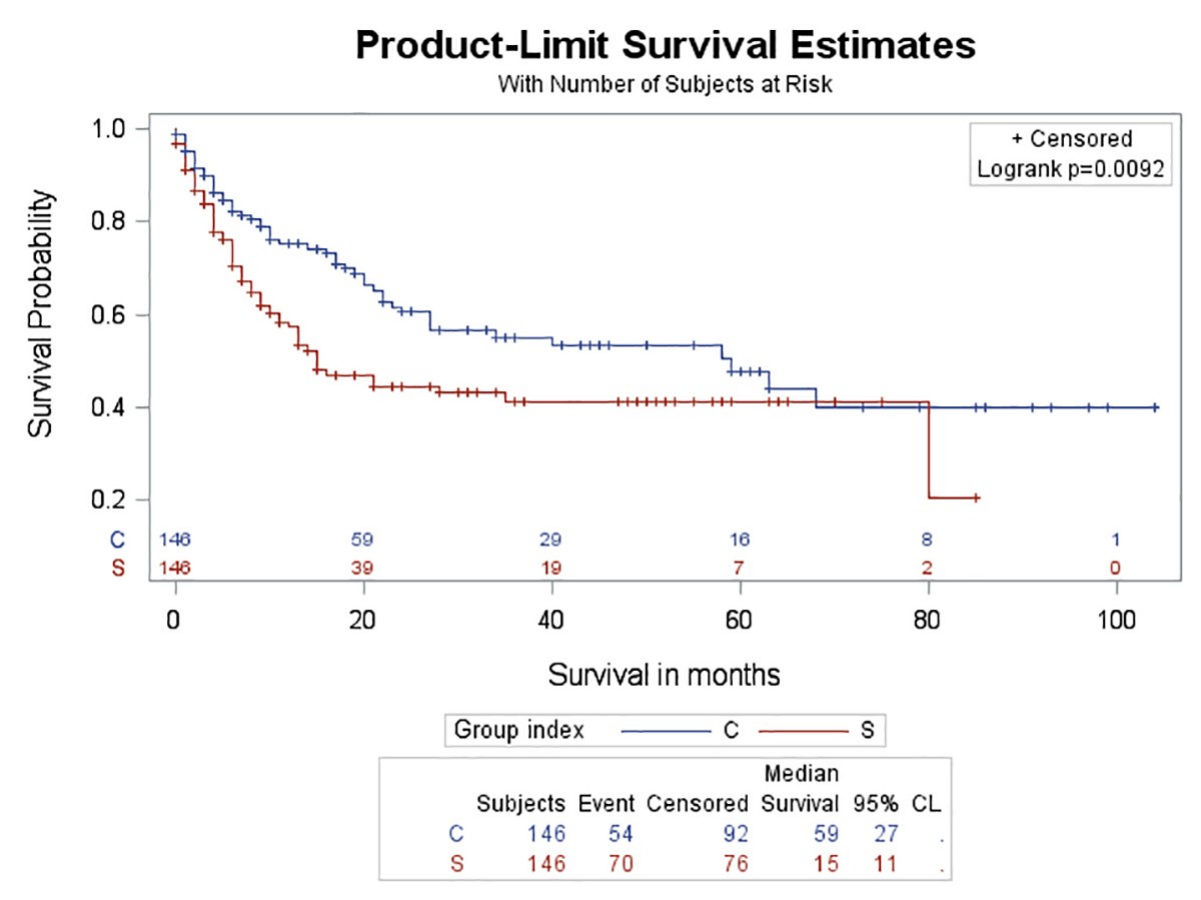
Survival curves for the study and control group. Survival curves for the study (S) group (red colour) and control (C) group (blue colour) (p = 0.0092).

Seventy out of 146 patients in the study group died during follow-up (48%). 54 out of 146 patients (37%) in the control group died during the follow-up. Median survival period was 15 months in the study group and 59 months in the control group. 5-year survival for the study group was 41% compared to 48% for the control group (log-rank test p = 0.0092) (Fig 4).

Men with lung cancer and ILD had overall lower survival rate compared to women (difference in 5 yr survival rate = 0.1701, log-rank p = 0.0381). No significant difference in survival was found between men and women with lung cancer only (difference in 5 yr survival rate = 0.0176, log-rank p = 0.429).

There was no significant difference in survival rate between those with emphysema and those without emphysema on CT in the study group (log-rank test, p = 0.6553) and in the control group (log-rank test, p = 0.1247).

**Fig 5:**
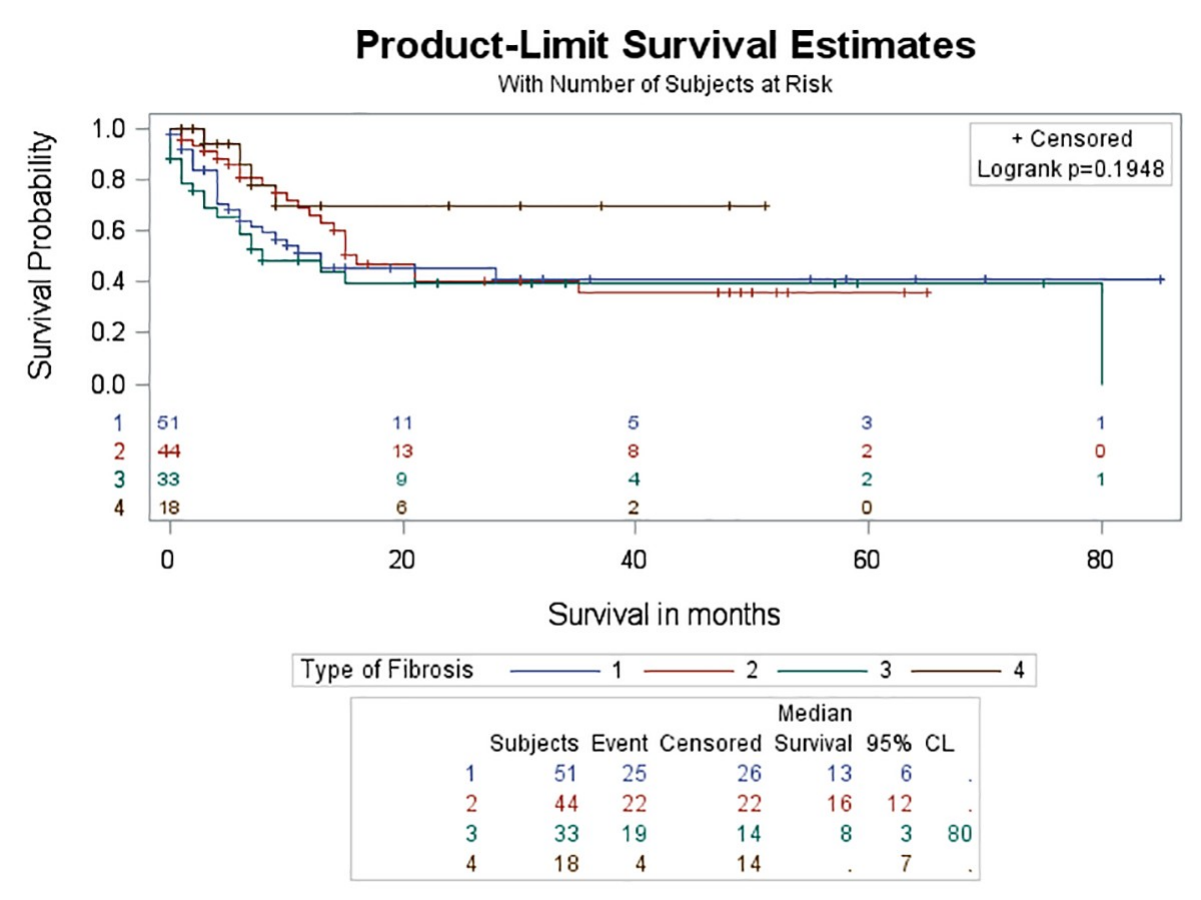
Survival curves between different types of ILD. There was no significant difference in survival rate between different types of ILD (log rank test, p = 0.195).

**Fig 6:**
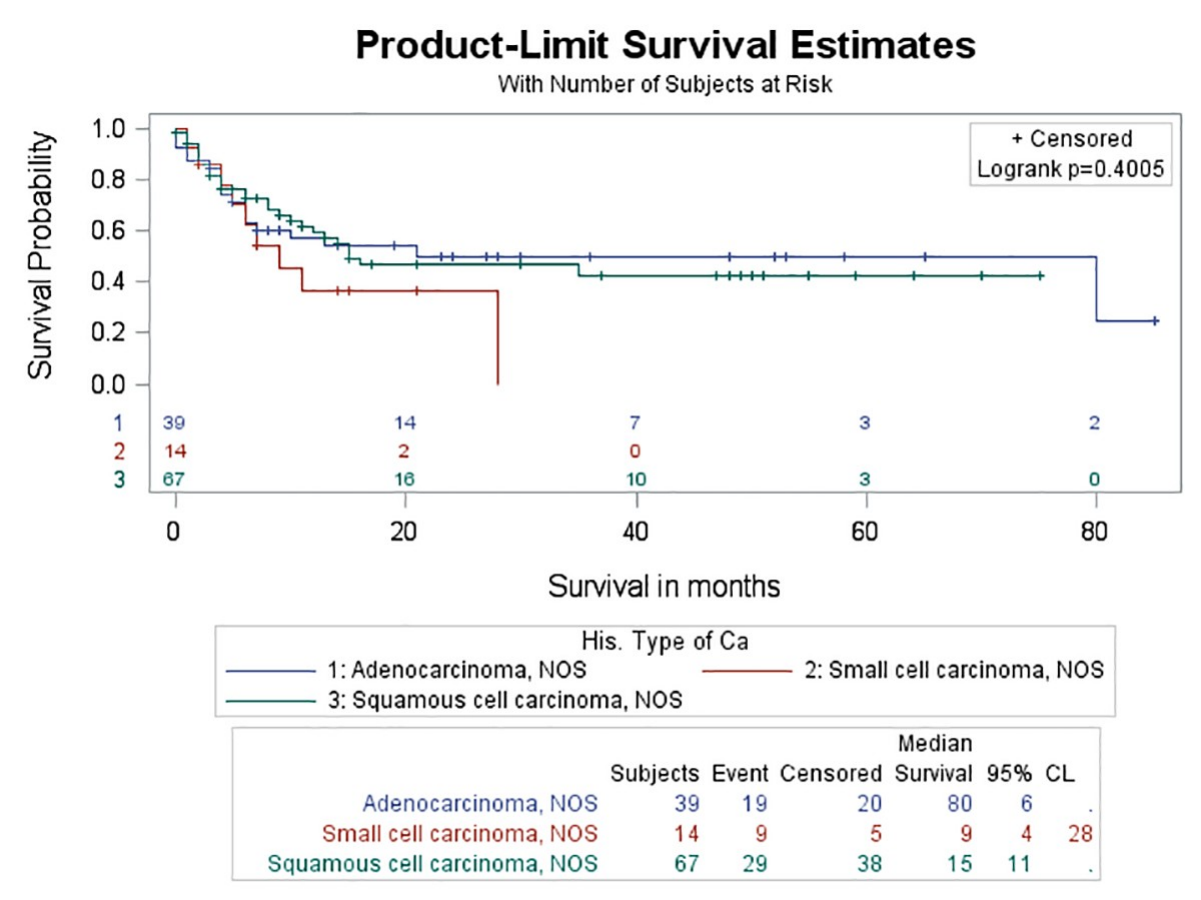
Survival curves between different types of lung cancer in the study group. There was no significant difference in survival rate between different types of lung cancer in the study group (log rank test, p = 0.4005).

**Fig 7:**
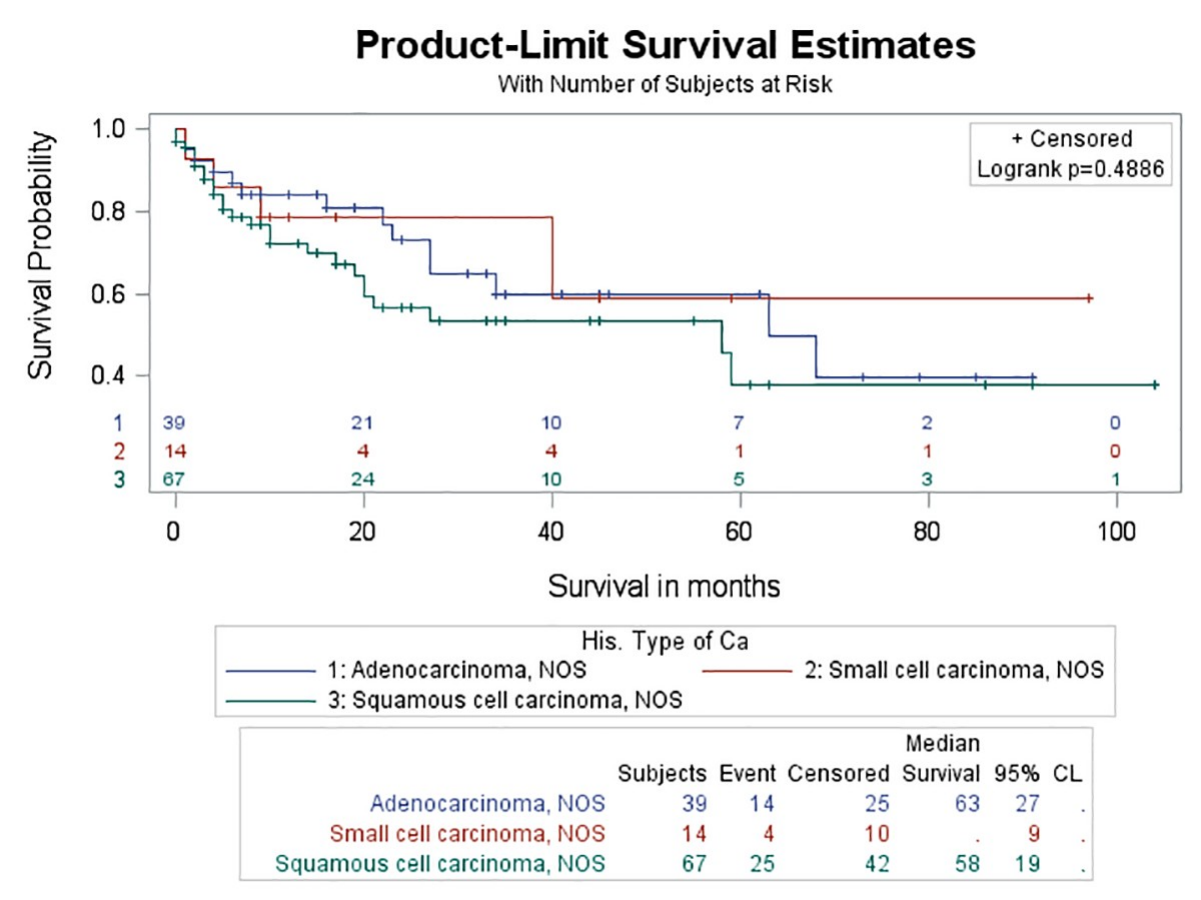
Survival curves between different types of lung cancer in the control group. There was no significant difference in survival rate between different types of lung cancer in the control group (log rank test, p = 0.4886).

No significant difference in survival rate was found either between the four different categories of ILD (log-rank test, p = 0.195) (Fig 5) or between the different histologic types of lung cancer among patients with lung cancer and ILD (log-rank test, p = 0.4005) (Figs 6 and 7). Although the probability for survival at 2 years was higher for adenocarcinoma (0.5019) compared to SCC (0.4669), yet this did not reach statistical significance (log-rank test, p = 0.9302).

**Fig 8:**
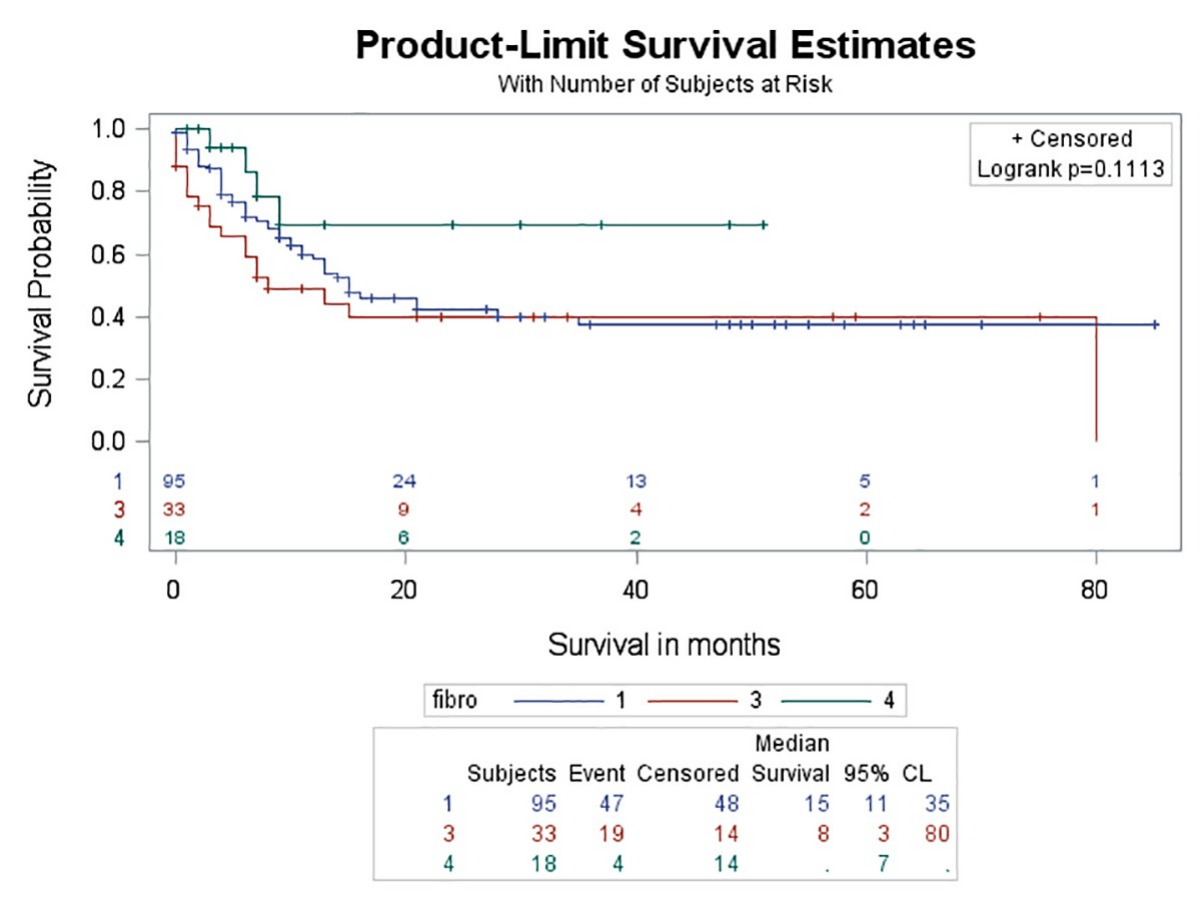
Survival curves between typical and probable UIP compared to other types of ILD. No significant difference was found in survival rate when combining typical UIP and probable UIP types and compared to other types of ILD (log rank test, p = 0.111).

After combining the first two types (UIP and probable UIP) into one category because of their high probability of having UIP pattern on pathology and comparing it with the other two remaining types (indeterminate for UIP and inconsistent with IPF), no significant deference in survival was found (log-rank test, p = 0.111) (Fig 8).

A cox proportional hazard model was used to estimate the hazard of death of patients with different risk factors. After variable selection, the type and extent of ILD, histologic type of cancer, presence of emphysema, smoking and treatment (yes or no) were removed from the model due to insignificant confounding effect and failure to improve the model fit. The final model included presence of ILD, clinical stage at diagnosis and age. The estimated hazard ratio for each risk factor by the adjusted cox model is shown in Table 4. All three factors were significantly associated with mortality. The hazard of death among the patients with ILD was 1.522 times that among the patients without ILD (95%CI, p = 0.029) when controlling for age and clinical stage. Patients with stage IA, IB, IIB and IIIA had a lower hazard of death or higher survival as compared to patients with stage IV. Finally, for every additional year of life, the hazard of death increased by a factor of 1.105 (95%CI, p = 0.042), when controlling for presence of ILD and clinical stage.

**Table 4:** Estimated hazard ratio from adjusted cox model.

Variable	Hazard ratio	95% CI	p-value
Presence of ILD	1.522	[1.043–2.222]	0.029*
Clinical stage			
Stage IA	0.121	[0.055–0.267]	<0.000***
Stage IB	0.270	[0.139–0.524]	0.000***
Stage IIA	0.000	[0.000-.]^a^	0.983
Stage IIB	0.273	[0.109–0.683]	0.006**
Stage IIIA	0.362	[0.222–0.589]	<0.000***
Stage IIIB	0.852	[0.468–1.550]	0.600
Stage IV	Reference	.	.
Age (per 5 years)	1.105	[1.003–1.216]	0.042*

*Note*: Asterisk means effect is significant at *p* < .05

* = *p* < .05

** = *p* < .001

*** = *p* < .0001.

a: due to a small sample of stage IIA patient, model fails to generate a robust hazard ratio estimation for this group of patients

Regarding treatment specifically, we found that in the unadjusted cox proportional hazard model, radiation treatment had a better outcome on survival of patients with lung cancer and ILD compared to chemotherapy or combined chemoradiotherapy. However the number of patients treated with chemotherapy was very low (13) and there was a significant number of patients censored either due to loss to follow up or because they did not receive any treatment, introducing instability and bias to the analysis. When we assessed whether the presence of treatment affected survival of patients with lung cancer and ILD we found that the hazard of death for those receiving treatment is 0.621 times that of those not receiving treatment (p-value 0.0314) indicating a positive outcome. However when treatment was added in the multivariate model, the model became unstable probably due to increased sparsity of the data regarding treatment and the imbalance of the censorship at the different types of treatment which again probably introduced instability and bias to the model.

## Discussion

Our study has shown that patients with concurrent lung cancer and ILD documented on CT have lower survival when compared to patients with lung cancer only, with median survival of 15 months versus 59 months and a 5-year survival of 41% vs 48%, respectively. Previous studies about lung cancer and ILD (the majority of the studies have focused on patients with IPF and CPFE) have also demonstrated lower survival and higher mortality rate among patients with lung cancer and ILD as compared to patients with lung cancer only or IPF only or emphysema without lung cancer. Gibiot et al [6], found median survival of 9 months for patients with lung cancer and ILD as opposed to 18 months for those with lung cancer only. Tzouvelekis et al [12], recently reported a median survival of 14 months in patients with lung cancer and IPF from the time of lung cancer diagnosis. Sekihara et al [13], reported 5 year-overall survival of 40% in patients with lung cancer and ILD as opposed to 72% in those with lung cancer only without ILD. A meta-analysis done by Koo et al [7], showed lower survival rate in patients with lung cancer and CPFE as compared to those with lung cancer only and those with lung cancer and emphysema. The median survival time was 20 months in patients with lung cancer and CPFE (5-year survival: 18%) and 53 months in those with lung cancer only without CPFE (5-year survival: 44%). Radiologists should be aware of the risk of ILD in patients with lung cancer and should appropriately classify the ILD pattern in their report to alert treating physicians. Physicians and healthcare providers should be aware of the risk of ILD in patients with lung cancer and should take it into consideration when selecting the appropriate treatment plan. In an era that surgical lung biopsy is not recommended for patients with a typical UIP CT pattern and the appropriate clinical context [8], and may be impractical in other cases of suspected ILD due to age, disease severity, other comorbidities or patient refusal it is very crucial to obtain prognostic implications from the CT documentation of ILD in patients with lung cancer [14, 15].

Official guidelines about the most appropriate chemotherapeutic plan for patients with IPF and lung cancer are lacking [16]. Chemotherapeutic regimens except for carboplatin have been reported to increase pulmonary toxicity in patients with IPF [12, 17]. Radiation therapy may also prove detrimental rather than beneficial for some patients with lung cancer and IPF. These patients may also have higher operative mortality and decreased 5-year survival compared to patients without IPF [12, 18].

Four different types of ILD were classified on CT according to the guidelines for the diagnosis of IPF [9]. No significant difference was found in the survival rate amongst the patient groups of lung cancer and the 4 different categories of ILD according to the diagnosis of IPF in both unadjusted and adjusted cox regression model. Similarly, Sekihara et al. reported no significant difference in the overall survival between patients with UIP pattern (41%) and those with non-UIP (possible UIP and inconsistent with UIP patterns) (41%) [13]. Maybe larger number of patients in future studies will be able to detect significant differences amongst the different types of ILD documented on CT.

Previous studies about lung cancer and ILD, have demonstrated that the majority of these patients are elderly smoker males [5, 19–27]. Our study also shows that 78% of the population are men. Approximately 82% of the study population were active or ex-smokers. Nezhad et al [5], in a recent meta-analysis about lung cancer and IPF found that 91% of patients enrolled in those studies were men and 91% were current and ex-smokers. Koo et al. in an earlier meta-analysis about lung cancer and CPFE reported that 97% of patients (620 patients in total) were men and 99% were heavy smokers [7]. The slightly lower percentage of smokers in our study is likely due to the significant number of patients with unknown smoking status (11%).

Squamous cell carcinoma (SCC) was the most common histologic type in our study (46%) followed by adenocarcinoma (27%). In two separate meta-analyses SCC was also found to be the most common histology with 38% and 42% followed by adenocarcinoma with 31% and 34%, respectively [5, 7]. A few studies have shown that, adenocarcinoma is the most common histologic type with SCC being second to adenocarcinoma [6, 25]. Gibiot et al. recently found adenocarcinoma to be the most common histologic type in 49 patients with lung cancer and concurrent ILD (47%) followed by SCC (20%) [6]. Survival in lung cancer patients with SCC—irrespective of concomitant ILD—is known to have worse survival compared to adenocarcinoma [28]. In our study although patients with SCC and ILD had worse survival compared to those with adenocarcinoma and ILD, yet this did not reach statistical significance. Probably this difference was altered by the concomitant ILD.

The majority of lung cancers in patients with concomitant fibrotic lung disease in our study were peripheral in location, within areas of fibrosis and were predominantly seen in the lower lobes, similar to other studies [29]. Stage IV was the most common stage present in 40% of cases compared to 47% of cases in other studies [6].

According to our knowledge this is the largest study to analyse patients with lung cancer and evidence of pulmonary fibrosis on CT classified according to the most recent recommendations for diagnosis of IPF published in 2018 [9]. Our study has shown that typical UIP CT pattern is the most common CT pattern to occur in association with lung cancer (35%) followed by probable UIP (30%), indeterminate for UIP (23%) and inconsistent with IPF (12%). In the study by Gibiot et al., where the authors also used the most updated recommendations for diagnosing IPF, they found probable UIP pattern to be the most common type seen in 32.7% of patients [6]. Typical for UIP CT pattern was seen in 20% of patients, indeterminate for UIP in 22% and inconsistent with IPF in 25% of patients. Unlike our study they have reported a much higher percentage of cases inconsistent with IPF (25% versus 12% in our study). Sekihara et al. analysed 2054 patients who underwent complete resection of stage IA-IIIA non-small cell lung cancer [13]. 5% of patients had ILD. They classified these patients using the older version of the recommended guidelines for diagnosing IPF published in 2011 categorised into 3 different types: UIP, possible UIP and inconsistent with UIP [30]. In their study the UIP type represented 75% of their population.

The majority of patients in our study had mild extent of ILD on CT (62%). Many studies have shown that patients with concurrent lung cancer and ILD have higher morbidity and mortality as compared to those with lung cancer without ILD. Kato et al. reported 1-, 3- and 5-year all-cause mortality rate of 54%, 79% and 93% respectively after lung cancer diagnosis in 70 patients with lung cancer and IPF [16]. Koo et al. on a meta-analysis for patients with lung cancer and CPFE found that 5% of patients developed acute exacerbation and up to 29% of patients (341 in total) developed postoperative complications [7]. More than 30% of our study group (lung cancer with ILD) had moderate degree of fibrotic lung changes (>25% involvement of lung parenchyma). This suggests that not only the presence but also the severity of fibrosis may have a strong effect on the morbidity and mortality in these patients.

80% of patients in the study group and 71% of patients in the control group had emphysema. In our study the presence and severity of emphysema did not affect survival amongst patients with lung cancer and ILD.

Treatment was found to improve survival of patients with lung cancer and ILD in our cohort. Although radiation therapy has been reported to adversely affect survival of lung cancer patients with ILD, in our cohort where the majority of patients had either radiation alone or combined chemoradiotherapy we found that radiation had a positive outcome on survival. However increased sparsity of the data regarding treatment, and imbalance of censorship at the different types of treatment increased instability of the multivariate model and treatment was not found to be significant and therefore valid conclusions cannot be drawn. Other studies have shown that diffuse ILD is a significant factor for radiation pneumonitis that can occasionally be fatal [31].

Our study has inherent limitations associated with its retrospective nature including missing data on follow-up of the patients, clinical staging, treatment and lack of histologic confirmation of the type of interstitial lung disease. Also, lack of standardized CT protocol may have affected the diagnosis and confidence of ILD classification on CT. Finally, sparsity of the data regarding treatment and the imbalance of the censorship at the different types of treatment may have introduced instability and bias to the model.

## Conclusion

Our study has shown that patients with lung cancer and evidence of fibrotic lung disease on chest CT have a significantly shorter survival compared to patients with lung cancer only. Although our results did not show significant differences in survival between the subtypes of ILD on CT, we recommend documenting the type and grading the severity of pulmonary fibrosis in all patients with lung cancer in order to appropriately tailor lung cancer treatment in conjunction with fibrotic lung disease therapy, which may otherwise go unmanaged. Taking into account the heterogeneous practices in patients with lung cancer and pulmonary fibrosis, we hope that future large prospective and randomized trials may improve our insight on the optimal management of this specific and vulnerable population.

## Supporting information

S1 Data (XLSX)
